# Differential radio-sensitivities of human chromosomes 1 and 2 in one donor in interphase- and metaphase-spreads after ^60^Co γ-irradiation

**DOI:** 10.1186/1756-6649-9-6

**Published:** 2009-06-16

**Authors:** Rupak Pathak, Adarsh Ramakumar, Uma Subramanian, Pataje GS Prasanna

**Affiliations:** 1 Armed Forces Radiobiology Research Institute, Uniformed Services University of Health Sciences, 8901 Wisconsin Avenue, Bethesda, MD 20889-5603, USA

## Abstract

**Background:**

Radiation-induced chromosome aberrations lead to a plethora of detrimental effects at cellular level. Chromosome aberrations provide broad spectrum of information ranging from probability of malignant transformation to assessment of absorbed dose. Studies mapping differences in radiation sensitivities between human chromosomes are seldom undertaken. Consequently, health risk assessment based on radio-sensitivities of individual chromosomes may be erroneous. Our efforts in this article, attempt to demonstrate differences in radio-sensitivities of human chromosome-1 and/or -2, both in interphase and metaphase spreads.

**Methods:**

Upon blood collection, dosimetry and irradiation were performed. Lymphocytes were isolated after whole-blood irradiation with ^60^Co γ-rays in the dose range of 0–5 Gy for both interphase, and metaphase aberration studies. Induction of premature chromosome condensation in interphase cells was accomplished using a phosphatase inhibitor, calyculin-A. Metaphase spreads were harvested from short-term peripheral blood lymphocyte cultures following colcemid arrest and using an automated metaphase harvester and spreader. Aberration analysis in both interphase and metaphase spreads were done using FISH.

**Results:**

In interphase, aberrant cell and aberration frequency involving chromosome 1 and/or 2 increased linearly with radiation dose. In metaphase, aberrations increased in a linear-quadratic manner with dose. Our studies ascertain that chromosome-2 is more radio-sensitive than chromosome-1 in both interphase and metaphase stages, albeit the DNA content of chromosome-2 is lesser than chromosome-1 by almost 10 million base pairs.

**Conclusion:**

Differences in radio-sensitivities of chromosomes have implications in genetic damage, chromosome organization, and chromosome function. Designing research experiments based on our vital findings may bring benefit to radiation-induced risk assessment, therapeutics and development of chromosome specific biomarkers.

## Background

**R**adiation-induced chromosome aberrations lead to a plethora of detrimental consequences at cellular level. Inter-chromosomal differences in radio-sensitivity may indicate various underlying differences such as organization of chromatin material or genetic damage etc,. Careful and systematic analysis of chromosomal radio-sensitivity reveals differential susceptibility of chromosome(s) for aberration induction. Further it has been recently reported that a specific set of periodic DNA motif in genomic DN A, influence human chromosome function via chromatin organization [1].

Chromosome size, in general, is proportional to DNA content. Pandita et al. [2] and Luomahaara et al. [3] support the general assumption that chromosome aberration induction by radiation, being proportional to DNA content. However, studies looking at the interrelationship between chromosome size, DNA content and radiation sensitivity are sparse and not well understood. Studies performed by Pandita et al. in G_0 _stage of human lymphocytes using premature chromosome condensation-fluorescence *in situ *hybridization has shown that chromosome size is directly related to aberration frequency [2]. Further Luomahaara et al. using a cohort of people, who sustained accidental exposure to radiation in Estonia in 1994, examined the distribution of radiation-induced break points in chromosomes 1, 2, and 4, in proportion to DNA content and localization of breaks along the chromosome. The studies revealed that yield of exchanges was equal to that expected from their DNA content both, in persons after accidental exposure and in vitro irradiated lymphocytes. Surprisingly, the break point location of complete exchanges was not random [3].

In contrast, many studies do not support the above notion. Some studies purport that chromosomes with higher DNA content are less susceptible to exchange aberrations as compared to smaller chromosomes [4-6]. The higher relative radiation sensitivity of smaller chromosomes may be due to non-random distribution of breakage points along the chromosomes as observed by Loumahaara et al. [3].

The implied radiation sensitivity of chromosomes is also influenced by aberration type studied. For instance, human chromosome-1 is more susceptible to translocations as compared to chromosome-2, while the latter is more prone to deletions [7]. The reasons for heterogeneous radio-sensitivities among chromosomes are not clearly understood and factors involved in differential chromosomal radio-sensitivity appear to be complex and to a small extent nebulous.

Our current study explores radiation-induced damages to human chromosomes-1 and -2 in un-stimulated peripheral blood lymphocytes in interphase, using a signal-transduction based PCC technique for concurrently measuring deletions and exchanges [8,9]. In addition, in lymphocyte metaphase spreads we analyze deletions and exchanges separately, using whole chromosome-specific DNA hybridization probes. Our studies demonstrate higher radiation sensitivity of chromosome-2 both in interphase- and metaphase-spreads. Our analyses and concurrent measurement of radiation-induced deletions and exchanges in interphase and metaphase cells for determining relative radio-sensitivities, adjudicate implications in health risk analysis.

## Methods

### Blood collection, dosimetry and irradiation

To avoid the effect of inter-individual differences in chromosomal radio-sensitivity if any, blood was drawn from a healthy female donor with no known history of ionizing radiation exposure beyond routine diagnostic exposures. After obtaining informed consent, 30 ml whole peripheral blood was collected by phlebotomy, into vacutainers containing sodium heparin as an anticoagulant (BD Biosciences, USA). The Uniformed Services University of the Health Sciences, Human Use Committee, Bethesda, MD, USA approved the informed consent form.

All irradiations were performed at room temperature in the bilateral field of AFRRI's ^60^Co gamma exposure facility in a specially fabricated array for blood vacutainer tube as previously published by Wilkins et al [10]. Whole blood was irradiated with ^60^Co γ-rays in the dose range of 0 to 5 Gy for both interphase PCC and metaphase assay with the average dose rate of 0.638 Gy/min.

### Lymphocyte isolation and culture condition

**A**fter 24 h of irradiation, which will allow restitution of initial breaks, lymphocytes were isolated from whole blood using a density gradient (Histopaque, Sigma, USA) and washed twice in phosphate buffer saline (PBS, pH 7.0, Gibco, USA) for studying chromosome-1 and -2 specific aberrations in interphase and metaphase spreads as below:

### PCC induction in interphase cells

Previously reported signal transduction method of PCC induction was used to study radiation-induced chromosome damage [8,9] with minor modifications. Briefly, lymphocytes were incubated in 5 ml of Marrow-max media (Gibco, USA) containing a phosphatase inhibitor, calyculin-A (Calbiochem, USA) at a final concentration of 50 nM (instead of okadaic acid as originally explained), a mitosis promoting factor p34*cdc2*/cyclin-B kinase (New England Biolabs, USA, 50 units/ml), and adenosine triphosphate (Sigma, USA, 100 μM) were used [8]. The culture was maintained in a 5% humidified CO_2 _incubator at 37°C for 3 h.

### Metaphase spread preparation

For metaphase spread preparation from the isolated lymphocyte cultures, lymphocytes cultures were initiated by the addition of 10 μl/ml of phytohemaglutinin (PHA, Gibco, USA) and incubated at 37°C. After 24 hours of culture initiation, 0.25 μg/ml colcemid (Gibco, USA) was added and cells were incubated for an additional 24 hours at 37°C before the collection of first-division metaphase spreads at 48 hours.

### Harvesting of chromosomes in interphase and metaphase

Following cell culture, interphase and metaphase chromosome spreads were harvested, using an automated metaphase harvester (Hanabi PII, ADSTEC Technologies, Japan), after treatment with a hypotonic solution (0.56% potassium chloride) and fixation in 1:3 acetic acid to methanol solution. Cell suspension was spread on clean glass slides, using a metaphase spreader (Hanabi, ADSTEC Technologies, Japan), at 37°C and 54% relative humidity.

### Fluorescence in situ hybridization (FISH)

The whole-chromosome DNA probes were used to label chromosome-1 and -2 following the manufacturer's recommended protocol (Cytocell Ltd., Cambridge, UK) for aberration analysis in both interphase and metaphase spreads. Freshly prepared slides were treated with 2× SSC (pH 7.0 for 2 mins) and then dehydrated in ethanol series (70%, 85%, and 100%), each for 2 mins. Probes for chromosome-1 and -2 were pre-mixed at a ratio of 3:7 and 10 μl of mixed probes was placed on to the slide, covered with 22 × 22 mm glass cover-slip (Corning, USA), sealed with rubber solution glue, which was allowed to dry at room temperature. Slides were then denatured at 75°C on a hotplate for 2 mins and hybridized overnight in CO_2 _incubator at 37°C. Slides were then washed in 0.4× SSC (pH 7.0) at 72°C for 2 mins after removing the cover-slips. Slides were again washed with 2× SSC. Finally, slides were counter-stained with 10 μl of DAPI (4, 6-diamidino-2-phenylindole) containing anti-fade and covered with a cover-slip.

Hybridized interphase cells and metaphase chromosomes were viewed with Olympus microscope under dark field. DNA probes for chromosome-1 and -2 conjugated with fluorochromes, Texas-red and FITC, respectively, appear red and green. Images were captured under 600× and 1000× magnification (BD Biosciences Bioimaging System, IPlab 4.0, USA). In metaphase spreads; translocation, dicentric and breaks involving only painted chromosomes are scored by adopting S&S classification [11]. Translocations are scored under two broad categories: reciprocal (represented by two bicolor monocentric chromosomes) and simple terminal 'one-way' translocation (simple incomplete/terminal exchanges I, II, and III as described by Simpson and Savage [11]). Among reciprocal translocation, two painted chromosomes are involved (chromosome 1 and 2); or one of the painted chromosomes (either chromosome 1 or 2) and another un-painted (DAPI stained chromosome) chromosome forms reciprocal exchanges. In case of reciprocal translocation between two painted chromosomes, we counted one aberration under each of chromosome -1 and -2. Complete reciprocal as well as two types of incomplete dicentrics (pattern IV and VI as originally described by Simpson and Savage [11]) are scored. Dicentrics involving chromosome-1 and -2 are counted as one aberration under each of chromosome-1 and -2. In interphase cells, whole chromosome DNA probe for chromosome-1 and -2 will produce 2 red and 2 green signals under normal condition of the applied FISH technique. Any extra signals for the respective painted chromosomes that are recorded has been inferred as aberrations resulting from either exchange or breaks.

### Statistical analysis

The data points for different types of aberrations were presented as normalized frequency. The background aberration frequency was subtracted from the irradiated aberration frequencies for each dose point. The standard errors of the frequency of each aberrations were calculated by v *a/A*, where *a*, represents the number under consideration and *A *is the total number of cells analyzed [12]. The data points were fitted with a linear equation, *y *= *αD *for intephase PCC assay, while for metaphase a linear-quadratic equation, *y *= *αD *+ *βD*^2 ^was used, where *y *is the yield of aberration, *D *is the physical radiation dose, *α *and *β *are constants. Curve fitting software (SigmaPlot 10.0, Systat Software, Inc., USA) was used to fit the data points. Comparison of curve fitting of dose-response curves were done by F-test using OriginPro-7.5 software (OriginPro 7.5, OriginLab Corp. Northampton, MA, USA). The statistical significance of the results were counter verified with ANNOVA.

## Results

### Aberrations involving chromosomes 1 and 2 in interphase cells

**Figure 1:**
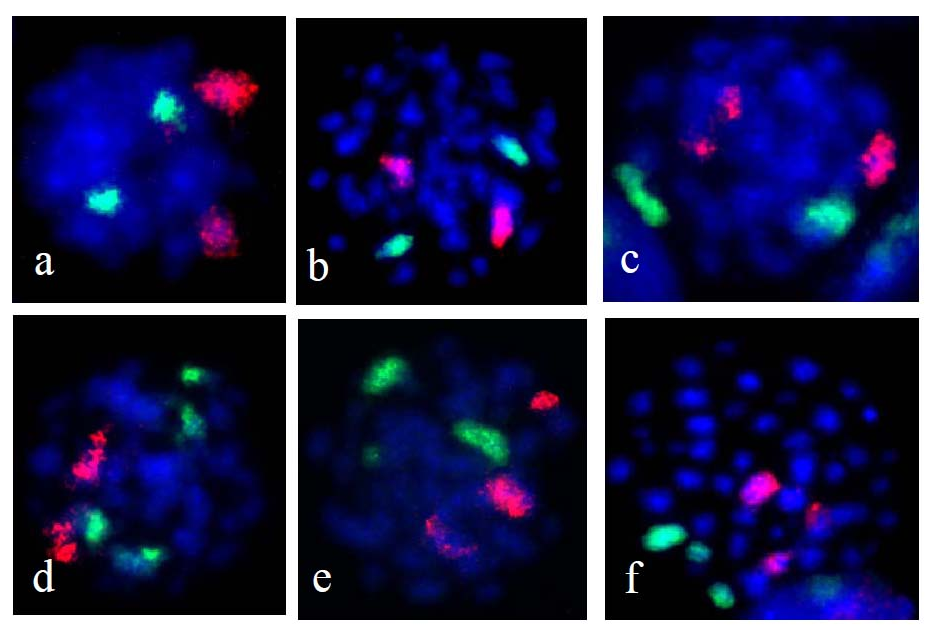
**Photomicrographs showing FISH painted human chromosome 1 (red) and 2 (green) in interphase lymphocytes induced by modified PCC method after ^60^Co γ-irradiation**. Normal cell producing two red spots and two green spots (a & b), aberrant chromosome 1 producing more than two red spots (c), aberrant chromosome 2 produced more than two green spots (d), while more than two green and red spots are seen while both the chromosomes bearing aberrations (e & f). Photographs were taken under 600× magnification.

**Figure 2:**
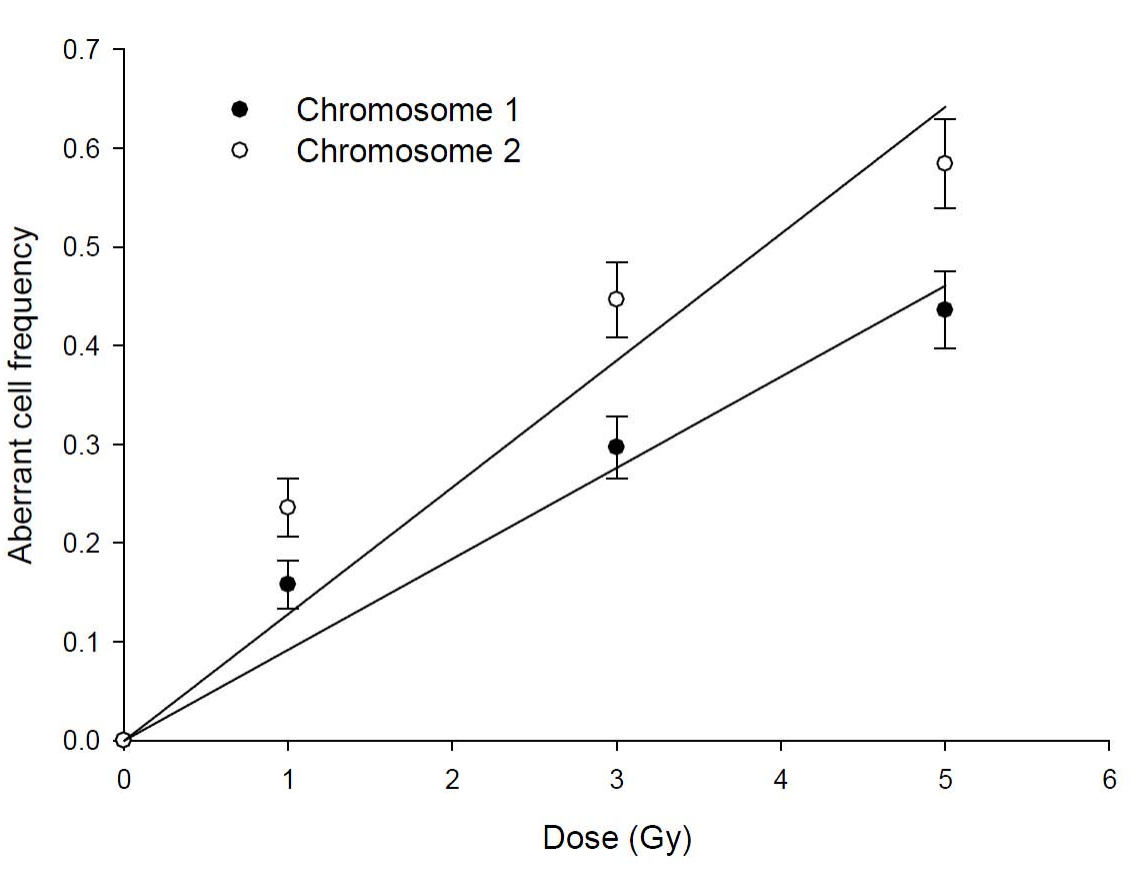
**Dose-response curves showing comparison of chromosome 1 aberrant cells and chromosome 2 aberrant cells in lymphocytes as detected by modified PCC method after exposure to ^60^Co γ-rays**. The aberrant cell frequency was fitted with a linear equation, *Y *= *áD*. The linear (*α*) coefficients of aberrant cell dose-response curves are 0.0922 ± 0.0071 and 0.1285 ± 0.0133 for chromosome 1 and 2, respectively.

**Figure 3:**
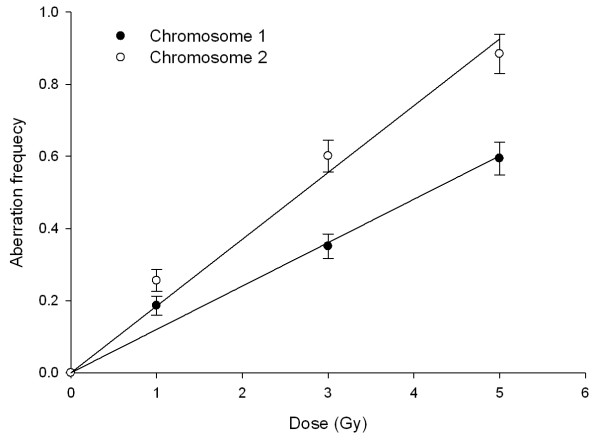
**Dose-response curves showing differential radio-sensitivity of human chromosome 1 and human chromosome 2 with respect to aberration induction in lymphocytes as detected by modified PCC method after exposure to ^60^Co γ-rays**. The aberration frequency was fitted with a linear equation, *Y *= *αD*. The linear (α) coefficients of aberration dose-response curves are 0.1203 ± 0.0065 and 0.1851 ± 0.0091 for chromosome 1 and 2, respectively.

Fig. 1 shows representative photomicrographs of FISH painted, prematurely condensed chromosomes 1 and 2, obtained by the modified PCC method, 24 h after ^60^Co γ-irradiation of peripheral blood lymphocytes. Table 1 shows the frequency of aberration in PCC spreads involving chromosome-1 and-2. Number of cells with aberrations in chromosome 2 is higher than number of cells with aberrations in chromosome 1 following irradiation across the dose range (Fig. 2). Fig. 3 shows the differences in radio-sensitivity between chromosomes 1 and 2 with respect to aberration induction. Linear trend in the dose-response curve is observed for both the chromosomes and chromosome-2 displays higher radio sensitivity.

**Table 1:** Aberrant cell and aberration involving human chromosome 1 and 2 after exposure to ^60^Co γ-rays as detected by FISH in interphase cells.

**Dose**	**Total cell**	**Chromosome-1**	**Chromosome-2**	**Chromosome-1**	**Chromosome-2**
**(Gy)**	**scored**	**Aberrant cell**	**Aberrant cell**	**Aberration**	**Aberration**
0	621	15	14	16	14
1	302	55	78	64	84
3	324	104	152	122	202
5	300	138	182	186	272

### Aberrations involving chromosomes 1 and 2 in metaphase

**Figure 4:**
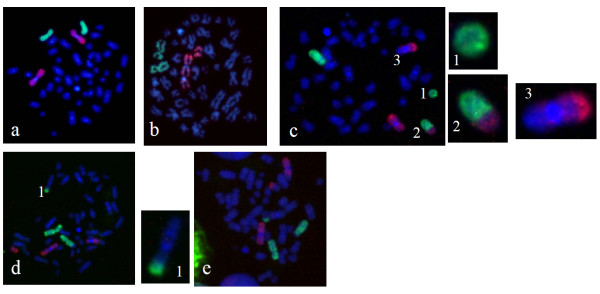
**Photomicrographs showing FISH painted human chromosome 1 (red) and 2 (green) in metaphase after exposure to ^60^Co γ-rays**. (a) Un-irradiated control cell showing two normal red chromosomes and two green chromosomes. (b) Chromosome 1 showing break. (c) Broken part of chromosome 2 (1 inset), translocation between chromosome 1 and 2 (2 inset) as well as chromosome 1 and unpainted chromosome (3 inset). (d) Reciprocal translocation between chromosome 2 and an un-painted chromosome (1 inset). (e) Both chromosomes 1 and 2 are involved in reciprocal translocation with un-painted chromosomes.

**Figure 5:**
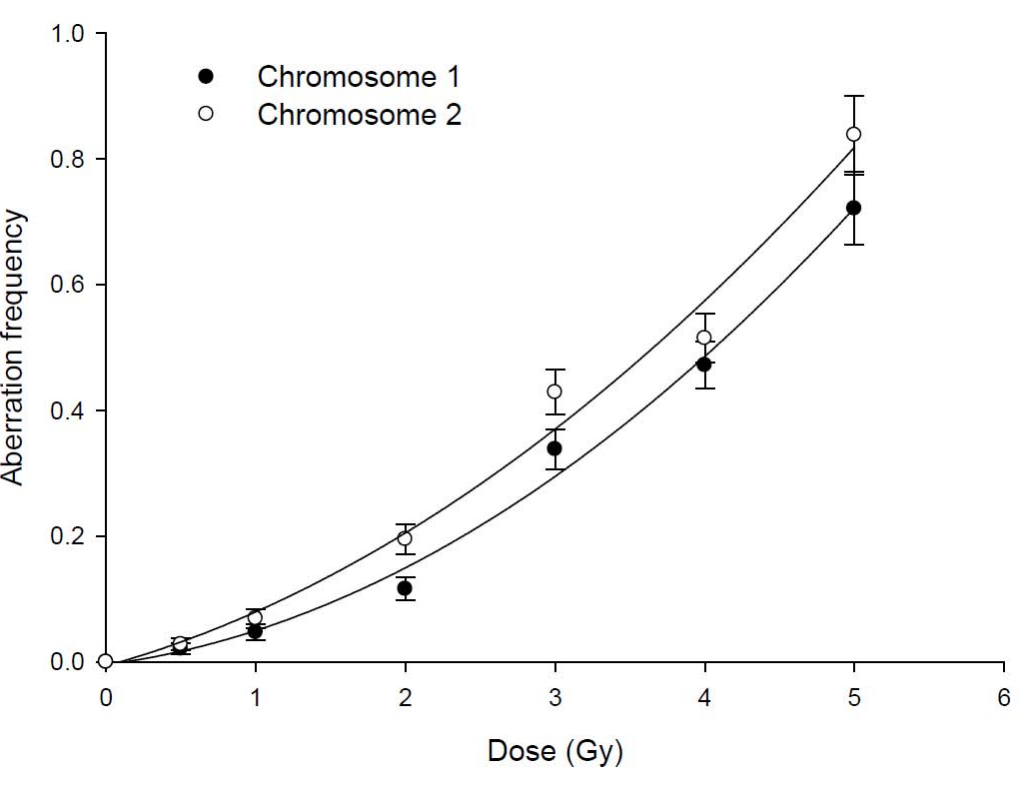
**Dose-response curves showing differential radio-sensitivity of human chromosome 1 and human chromosome 2 with respect to aberration induction in lymphocytes as detected by metaphase FISH analysis after exposure to ^60^Co γ-rays**. The dose-effect relationship is best described by *Y *= *α D *+ *β D*^2^, where *α *and *β *are the coefficients of the fitted curve. The *α *coefficients of aberration dose-response curves are 0.0315 ± 0.0235 and 0.0663 ± 0.0361 for chromosome 1 and 2, and *β *coefficients of aberration dose-response curves are 0.0228 ± 0.0046 and 0.0197 ± 0.0071 for chromosome 1 and 2, respectively.

Chromosome-1 and -2 aberrations are also studied in metaphase spreads using whole chromosome DNA probes. Fig. 4 shows a representative photomicrograph of metaphase chromosomes after FISH. We scored different types of translocations and breaks produced in both the chromosomes. Fig. 5 shows comparison of aberrations between chromosome-1 and -2 after irradiation. The increase in aberration frequency is linear quadratic with dose for both chromosomes and chromosome-2 exhibited higher sensitivity, also in metaphase spreads similar to observations in interphase.

Table 2 shows spectrum of aberrations in metaphase involving chromosome-1 and -2 after exposures to ^60^Co γ-rays. Different types of aberrations increased with increasing radiation dose. The frequency of total translocations is higher than breaks and dicentrics; and the frequency of terminal translocations is higher than reciprocal translocations.

**Table 2:** Spectrum of aberrations involving chromosome 1 and 2 after exposure to ^60^Co γ-rays as detected by FISH in metaphase cells.

**Dose**	**Total cell**	**Reciprocal**	**Terminal**	**Total**			**Total**	**Chromosome-1**	**Chromosome-2**
**(Gy)**	**scored**	**Translocation**	**Translocation**	**Translocation**	**Dicentric**	**Break**		**Aberration**	**Aberration**
0	674	1 (1)	2	3	0 (0)	3	6	4	3
0.5	372	3 (2)	7	10	1 (1)	8	19	10	12
1	340	10 (2)	15	25	2 (2)	12	39	18	25
2	361	33 (5)	42	75	9 (1)	26	110	44	72
3	337	67 (9)	97	164	14 (3)	72	250	116	146
4	339	80 (17)	117	197	30 (6)	88	315	162	176
5	216	60 (16)	141	201	36 (8)	78	315	157	182

Values in parenthesis reflect the number of reciprocal or dicentric chromosomes formed between chromosome 1 and 2.

## Discussion

Since radiation-induced chromosome aberrations lead to calamitious consequences at cellular level, via genetic damage, chromatin organization, and chromosome function [1], systematic study of differences in inter-chromosomal radio-sensitivities is imperative. The relationship between chromosome size or DNA content and its effect on radio-sensitivity is debatable [2-6]. Our study was aimed to characterize the relative radio-sensitivities of human chromosome-1 and -2, both in interphase- and metaphase-spreads and thereby broadly understand differences in radiation-induced genetic damage, chromatin organization, and chromosome function. Chromosome-1 spans about 279 million nucleotide base pairs and chromosome-2 about 251 million base pairs [12] but, the former seems to contain approximately 3-times more genes [13]. Imperatively, differences in radio-sensitivity between these chromosomes will result in differences in genetic consequences.

The difference in radio-sensitivities between chromosome-1 and -2 is evident from the studies of Fernadez et al., in metaphase spreads [14] and in interphase cells [15]. Our laboratory earlier developed a simple and rapid signal transduction method to study radiation-induced specific chromosome damages directly in un-stimulated peripheral blood lymphocytes. This approach allowed comprehensive identification and quantification of radiation-induced damages, which represents breaks and/or translocations in interphase cells [8,9]. In the present study, we further developed this method to be able to simultaneously measure damages in two chromosomes enabling studies on inter-chromosomal differences in radiation sensitivity directly in un-stimulated peripheral blood lymphocytes and compare these observations in metaphase spreads.

We observed a linear dose-response in radiation-induced aberration frequency and aberrant cells for both the chromosomes in interphase cells, confirming our previous results on chromosome 1 damage [8]. The *p *and *r*^2 ^values of the linear dose-response curves for aberrant cells with chromosome-1 and -2 indicated statistically significant (*p, 0.001 *and *0.002 *and *r*,^2 ^0.94 and 0.90, respectively) goodness of fit with this model. The observed significant difference (*p *<*0.05*) between dose-response curves for chromosome-1 and -2 aberrant cells indicates a difference in radio-sensitivity. Further, chromosome-2 aberrant cells were higher indicating a higher radio-sensitivity, which is evident in the linear component of the model as seen by a significant difference in slope (Fig. 2).

We further characterized the differences in radio-sensitivity between chromosome-1 and -2 by measuring aberration frequencies. Linear dose response curves as seen for aberrant cells reflected in aberration frequencies for chromosome-1 and -2. We plotted dose response curves with a linear equation by subtracting the background aberration frequency for specific chromosomes from radiation-induced aberration frequency. The *p *values of *0.0004 *and *0.0003 *as well as *r*^2 ^values of 0.98 and 0.98, respectively, for chromosome-1 and -2 aberration frequencies reflect significant goodness of fit with the model. We noticed higher aberration frequency for chromosome-2 as evident from the significant difference (*p *<*0.05*) in dose response curves reflecting higher radio-sensitivity of chromosome-2.

Using whole chromosome DNA hybridization probes we measured fragments and/or translocations involving human chromosome-1 and -2 in metaphase spreads. Linear-quadratic dose-response model is routinely used by others to study radiation-induced aberrations involving specific chromosome(s) [8,16]. Our observations that linear-quadratic increase in aberrant cell as well as total aberrations with radiation doses corroborates the observations of Fernandez et al. [14] on translocations and dicentrics involving chromosome-1 and -2 after exposure to 100 kVp X-rays. Linear-quadratic dose-response curves were also seen for translocations involving chromosomes 2, 8, and 14 after exposure to ^60^Co γ-rays [6].

Our studies in metaphase spreads indicate a lower aberration frequency in chromosome-1 similar to observations in interphase cells. Comparison of two dose-response curves demonstrated a significant difference (*p *<*0.05*). Previously, Wojcik and Streffer [7] observed a lower frequency of acentric fragments involving chromosome-1 than -2 after irradiation with 1 Gy of X-rays. However, at a higher dose of 2 Gy the over-expression of acentric fragments (breaks) was only found in one experiment.

We observed dose-dependent increase in the frequency of different aberration types involving human chromosome-1 and/or -2 in metaphase cells. The stable translocation frequencies involving both the chromosomes were higher than breaks. Earlier, Grigorova et al. [16] reported lower frequencies of acentric fragments (breaks) compared to translocations involving chromosome 2, 3, 8, X, and Y in Chinese hamster splenocytes exposed to X-rays and neutrons.

The frequencies of stable translocations were higher compared to dicentrics in our studies involving chromosome-1 and/or -2, similar to the observations of others [17-20]. Translocation frequency was significantly higher than dicentrics in Chinese hamster splenocytes exposed to both low LET X-rays and high-LET neutron beams [16]. Higher relative frequencies of stable translocations compared to dicentrics was also observed in interphase cell as measured by the PCC-FISH technique involving human chromosome 8 after X-irradiation [21].

Our study revealed that human chromosome-2 is more prone to aberration induction compared to chromosome-1 both in interphase and metaphase cells though the DNA content of chromosome-2 is approximately 2.4% less than that of chromosome-1. The apparent reasons for differences in radio-sensitivities between these two chromosomes may be due to a difference in spatial organization in the nucleus. Recently Branco et al. showed a difference in spatial organization of human chromosome territories as indicated by the average radial positions. The average radial position of chromosome-2 is higher than chromosome-1 both in resting and PHA stimulated human lymphocytes [22], which we strongly believe to be involved in bringing out the differential radio-sensitivity among chromosome 1 and 2.

In general interphase cells display more number of aberrations per unit dose compared to metaphases (Tables 1 and 2). However, this is influenced by: (i) a dose-dependent increase in aberration yield in both interphase- and metaphase- spreads, but the degree of difference is not dose-dependent because of a differential saturation of aberrations and elimination via cell death. This is evident from the apparent similar yield in aberrations, particularly at 5 Gy. (ii) In our studies we used whole chromosome DNA probes for both interphase- and metaphase-spread analyses; therefore, it is prudent to expect loss of some signal in interphase cells, since the DNA probes are region specific and bind along the length of whole chromosome, where DNA is not completely condensed akin to metaphases.

The differential radio-sensitivity between chromosome-1 and -2 may also be linked with the differences in the GC and AT base pairs among them and particularly in the regions of GC or AT richness [23,24]. We are currently investigating such differences at the molecular and genomic level that contribute to the radio-sensitivity among chromosomes and the effect of ionizing radiation and repair mechanisms on the chromosomes.

Various factors such as inter individual variability, DNA content, genetic background etc., can influence the radio sensitivity of individual chromosome [6,25]. *In lieu *of potential inter-individual variability as well as genetic background, affecting the aberration yield involving specific chromosomes, we have deliberately chosen only one donor for our experiments, so that that profound difference among chosen chromosomes would surface. This would abet the design of more targeted studies keeping specificity and sensitivity and underlying variation among genetic population of interest in mind. It is difficult to generalize or draw any systematic and precise conclusion on genetic contribution in context of the entire genomic population by using one donor. Never-the-less, the work presented here should emphasize the importance and need for tailored assessment of health risks for individuals, based on relative individual chromosomal radio sensitivities, given differences among chromosomes may exist among individuals and in populations. Further while gene mapping for health risks for instance like cancer, is complex and evolving using chromosomal changes may be a simpler alternative approach with additional emphasis on sensitivity and specificity metrics. The differential radio-sensitivity with respect aberration induction will affect the chromosome function via change in the genetic organization especially translocations. Detection of chromosome translocations assists in diagnosis, treatment and prognosis of many blood related cancer and childhood sarcoma [26]. For instance, a specific translocation between chromosome-1 and -13 results in alveolar rhabdomyosarcoma [27]. Similar interesting studies can be designed and investigated using the differences we have proposed in our current paper.

## Conclusion

The differences in radio-sensitivities of chromosomes have implications in genetic damage, chromosome organization, and chromosome function. The present study revealed that human chromosome- 1 and -2 show differences in sensitivities to ionizing radiation both in interphase cells and metaphase spreads. The dose-response curves for aberrations were linear and linear-quadratic in nature in interphase and metaphase spreads, respectively. Study of aberration spectrum in metaphase spreads involving human chromosome-1 and/or -2 demonstrated that frequency of translocation was higher than dicentrics as well as acentric fragments. Human chromosome-2 exhibited higher radio-sensitivity. The authors conclude with a preamble that further studies of larger scale needed to be taken and our current study may help in accelerating such efforts leading to a new focus arena, with broad implications in radiobiology, radiation therapeutics and cancer research by opening avenues for identification of chromosome specific biomarkers.

## Abbreviations

(AFRRI): **A**rmed Forces Radiobiology Research Institute; (DSB): Double strand breaks; (FISH): Fluorescence *in situ *hybridization; (HPBL): Human peripheral blood lymphocyte; (PCC): Premature chromosome condensation

## Competing interests

The authors declare that they have no competing interests.

## Authors' contributions

RPwas instrumental in designing the entire study, collection of data and analysis. USparticipated in carrying out lymphocyte isolation and irradiation of samples. ARwas instrumental in manuscript preparation, interpretation and analysis of data and key revising it critically for its intellectual content. PGSconceptualized and critically evaluated the entire study design, collected data and performed the study including funding. All authors have read and approved the final contents in the manuscript.

## Pre-publication history

The pre-publication history for this paper can be accessed here:


